# Maternal mortality ratio in selected rural communities in Kebbi State, Northwest Nigeria

**DOI:** 10.1186/s12884-018-2125-2

**Published:** 2018-12-21

**Authors:** Usman Gulumbe, Olatunji Alabi, Olusola A. Omisakin, Semeeh Omoleke

**Affiliations:** 1 Department of Mathematics and Statistics, Federal University, Birnin-Kebbi, Kebbi State Nigeria; 2 Department of Demography and Social Statistics, Federal University, Birnin-Kebi, Kebbi State Nigeria; 3 Immunisation, Vaccines and Emergencies Unit, World Health Organisation, Abuja, Nigeria

**Keywords:** Maternal mortality, Sisterhood method, Kebbi state, Northwest Nigeria

## Abstract

**Background:**

Maternal mortality remains a topical issue in Nigeria. Dearth of data on vital events posed a huge challenge to policy formulation and design of interventions to address the scourge. This study estimated the lifetime risk (LTR) of maternal death and maternal mortality ratio (MMR) in rural areas of Kebbi State, northwest Nigeria, using the sisterhood method.

**Methods:**

Using the sisterhood method, data was collected from 2917 women aged 15–49 years from randomly selected rural communities in 6 randomly selected local government area of Kebbi State. Retrospective cohort of their female siblings who had reached the childbearing age of 15 years was constructed. Using the most recent total fertility rate for Kebbi State, the lifetime risk and associated MMR were estimated.

**Result:**

A total of 2917 women reported 8233 female siblings of whom 409 had died and of whom 204 (49.8%) were maternal deaths. This corresponds to an LTR of 6% (referring to 11 years before the study) and an estimated MMR of 890 deaths/100,000 live births (95% CI, 504–1281).

**Conclusion:**

The findings provide baseline information on the MMR in rural areas of the State. It underscores the need to urgently address the bane of high maternity mortality, if Kebbi State and Nigeria in general, will achieve the health for all by year 2030 as stated in the Sustainable Development Goals (SDGs).

**Electronic supplementary material:**

The online version of this article (10.1186/s12884-018-2125-2) contains supplementary material, which is available to authorized users.

## Introduction

Maternal mortality remains a topical issue, and it is often used as one of the indicators for assessing the health status of a population as well as classifying countries into “developed” and “developing” countries. According to the tenth revision of International Classification of Diseases (ICD-10), pregnancy-related death is death of a woman during pregnancy or within 42 days of the termination of such pregnancy without considering the cause of death [1]. The occurrence of maternal death has huge consequences not only for the family but also the society in general. As pointed out by Ogunjimi et al. [2], survival of infants and under-five children closely depends on the survival of the mother. They opined that maternal mortality could reduce the life span and life chances of young children because children whose mothers are dead would lack daily care and often become delinquent.

It is clearly evident from literature that Nigeria has persistently ranked as one of the countries with most appalling maternal mortality ratio (MMR), accounting for an estimated 14% of global maternal deaths [3]. Reducing the high level of maternal mortality to an acceptable level agreed upon by the global community is a collective as well as individual national government’s efforts. This quest to achieve an acceptable maternal mortality level is often hampered by lack of accurate and reliable data to monitor progress. In most developing countries, vital registration is non-existent. Therefore, many rely on population census and survey ─ which oftentimes are not conducted at regular interval.

In Nigeria, Northern regions ─north central, north east and north west ─ have the highest MMR with estimated maternal mortality of more than 1000/100,000 live births in some of the states [4–6]. For example, Kebbi State, one of the north western states, has one of the worst reproductive health indices in Nigeria. More than two-third (71%) of pregnant women in the state never attended antenatal care; 91% of the women delivered at home; less than 10% had skilled birth attendance during the most recent birth, among others [1].

In recognition of this appalling statistics, the Nigeria Minister of Health recently inaugurated the “Task Force on Accelerated Reduction of Maternal Mortality in Nigeria” and stated that six northern States including Kebbi were among States with the highest burden in Nigeria [7]. Prior to this, the State Government of Kebbi and Adamawa in 2013 accessed a grant of 30 million Euros (six billion naira) from The European Union Commission to help reduce MMR in the two states [8]. Despite these, achieving a drastic reduction in MMR in Kebbi State remains a challenge. The effort is further hampered by the low utilization of health facilities for any kind of health services including child birth, low literacy level and strong socio-cultural dictates which prevent documentation of mortality events [9]. In a bid to overcome these challenges associated with evidence for policy formulation, an indirect method of estimating MMR is often resorted to in most developing countries.

The use of indirect technique of estimation of MMR, such as the sisterhood methods, is best suited for settings like Nigeria where vital statistics are deficient or non-existent [10]. Sisterhood method requires a smaller number of respondents, data collection is retrospective, simple, quick and based on information about maternal deaths among sisters of the respondents [5]. However, the method does not measure current level of MMR estimate for the current year of the survey but provides retrospective estimate for 10–12 years preceding the survey. The method has been widely used in some States in Nigeria. For instance, Doctor et al. [4] estimated MMR in four states of Jigawa, Katsina, Yobe and Zamfara in Northern Nigeria as 1271 deaths per 100,000 live births. In Plateau State, MMR of 905 per 100,000 live births was estimated using sisterhood method [11]. Also in Kaduna State, a MMR of 1400 deaths per 100,000 live births was estimated using sisterhood method [12]. However, there has not been any such study in Kebbi State.

In addition, the recent assertion by the Honourable Minister of Health underscores the need for a baseline study in Kebbi State to estimate MMR in a bid to measuring her progress towards the achievement of Sustainable Development Goal number three. Furthermore, the recent influx of non-governmental organisations (NGOs) working to improving maternal and child health in Kebbi State also necessitates the generation of baseline data on MMR situation in the State to monitor and evaluate the contributions of the NGOs towards the reduction.

This study sets out to estimate the lifetime risk (LTR) of maternal death and the associated MMR in selected rural villages in Kebbi State. Thus, the study will provide policy makers, government and stakeholders with baseline information on the MMR situation in the State and bring to fore the need to urgently address the scourge of high MMR in Kebbi State and in general, if Nigeria will achieve the health for all by year 2030 as stated in the Sustainable Development Goals (SDGs).

## Methods and materials

### Study area

Kebbi State, northwest Nigeria is a unique state due to its geographical location bordering two countries of Niger and Benin, and three other northern Nigerian States of Sokoto, Niger and Zamfara- with similar poor maternal and child health indicators. The study was carried out in randomly selected villages across the three senatorial zones of Kebbi State, northwest Nigeria. A total of six Local Government Areas (LGA) were randomly selected from the twenty-one LGAs in Kebbi State (i.e two LGAs were selected from each Senatorial Zone). Within the LGAs, rural communities were randomly selected.

Kebbi State is unique for this kind of study due to its position as one of the six states in the country with the highest number of maternal deaths due to inefficient and inadequate social amenities and health infrastructures- characteristic of a typical rural northern setting. There is also low rate of out-of-wedlock births despite sexually active population, which may suggest a high rate of abortion─ a factor that may influence the estimate─ in the study area [13].

### Data collection

The study adopted a retrospective cross-sectional design which utilizes quantitative data collection methods. The quantitative data was a cross-sectional household survey conducted in August, 2017. Six LGAs were randomly selected from the twenty-one LGAs from the three Senatorial zones in the State. Within the selected LGAs, a number of communities were randomly selected with a target sample size of an average of 500 (translating to 3000 women aged 15–49 in all the six LGAs) women of reproductive age from each LGA as proposed by Graham et al. [10].

Ethical clearance for the study was obtained from the research and ethics committee of the Federal University, Birnin kebbi. The ethics committee approved the use of verbal consent obtained from respondents before the commencement of the survey. The need to obtain parental consent for respondents under age of 16 was further waived by the committee. In each of the community sampled, approval was first sought from the district heads and village heads (*mai-anguwar)* before commencement of field work. During the field work, verbal consent was obtained from each respondent before administering questionnaires. There was a response rate of 97.2% during the survey as few of the respondents did not give their consent. Thus, 2917 sisters of childbearing age were interviewed.

Field team was trained on the use of electronic data collection methods and interview skills. During the training, the team was trained using questionnaire translated to Hausa and, back to English for ease of comprehension and administration in Hausa language on the field. Role plays were conducted in Hausa language during the training. An electronic-based sisterhood questionnaire (see Additional file 1) was administered on randomly selected women aged 15–49 years. Respondents were asked of the total number of sisters they had (born to the same mother). Out of these female siblings, the number exposed to the risk of childbearing i.e., married, was recorded (since child bearing is only accepted within the confines of marital union and there is high rate of early marriage in the study area). Specifically, the following questions were asked: “of the maternal sisters exposed to the risk of childbearing, how many of them were alive and how many were dead? Of the dead sisters, how many died while they were pregnant, or during childbirth, or during the six weeks after the end of pregnancy?” Age at death and place of death of sisters who died as a result of maternal death was also collected for each of the sisters.

The use of electronic-based data collection and recording of geo-coordinates of all household sampled afforded real time data analysis as well as minimising data entry error and data falsification. Data was checked for consistency and quality assurance. The data verified was downloaded from the dedicated surveyCto server into the computer using Excel spreadsheet. The data was exported to MMR calculation template developed for the purpose to generate the LTR and the associated MMR. Migration, which is an important factor in completeness of data was very minimal in the selected communities as most migration of women of reproductive age are due to marriage and intra-state. Thus, respondents accounted for all the female siblings with the help of consistency checks built into the e-based questionnaire.

### Analysis

Sibling history data was analysed and disaggregated into five-year age group. Number of maternal sisters exposed to the risk of maternal death and duration of exposure to the risk for each age group was computed by multiplying the number of reported sisters by an adjustment factor for the age groups. The lifetime risk (LTR) of maternal death was calculated using the total number of maternal deaths divided by the estimated total number of sisters exposed. Estimate of total fertility rate (TFR) of 6.7 for Kebbi State was obtained from the 2013 Nigeria Demographic and Health Survey (NDHS). MMR was computed using the formula: MMR = 1 – [(1-LTR)*(1/TFR)] and 95% Confidence Interval (CI) was obtained using formula for calculating CI outlined in Hanley et al., (1996).

## Result

Table 1 shows the socio-demographic characteristics of respondents interviewed. Of the 3000 women of child bearing age interviewed, 83 (2.8%) did not consent to be interviewed yielding a response rate of 97.2%. Highest number of respondents that consented were in 25–29 age group. As observed, more than half of the respondents had Islamic education (57.9%) while 3 in 10 of them had no formal education. Majority (96.3%) of the respondents were however married as at the time of the study.

**Table 1:** Respondents socio-demographic characteristics

Respondents characteristics	Frequency *N* = 3000	Percentage
Local Government		
Birnin kebbi	582	19.4
Maiyama	429	14.3
Fakai	310	10.3
Shanga	500	16.7
Augie	720	24.0
Bunza	459	15.3
Consent granted?		
Yes	2917	97.2
No	83	2.8
Age group		
15–19	494	16.9
20–24	666	22.8
25–29	711	24.4
30–34	412	14.1
35–39	314	10.8
40–44	202	6.9
45–49	118	4.1
Level of Education		
No formal education	907	31.1
Islamic education	1691	57.9
Primary education	162	5.6
Secondary education	139	4.8
Post secondary education	18	0.6
Marital Status		
Single	42	1.4
Married	2809	96.3
Divorced	34	1.2
Separated	5	0.2
Widowed	27	0.9

Table 2 shows the sisters’ vital status by five-year age group and the computation of LTR and MMR for the study area. In all, a total of 2917 women of child bearing age reported 8233 maternal sisters. Of the 8233 female siblings, 409 were reported dead and 204 of the dead sisters died a pregnancy and childbearing related death (maternal death). Lifetime risk of maternal death was 6% or 1:16 and TFR of Kebbi State was 6.7, thus a MMR of 890 deaths/100,000 livebirths (95% CI, 504–1281) was estimated for the State. The approximate time reference for the MMR estimate was the year 2006 corresponding to 11 years before the interviews.

**Table 2:** Responses of 2917 respondents about their sister’s vital status and the lifetime risk of maternal death in Kebbi State

Age group of respondent (years)	No. of respondents (%)	No. of maternal sisters ever married	No. of sisters who died (%)	No. of maternal deaths (%)	Adjustment factor	Sisters exposed (Col3×Col6)	Lifetime risk (Col 5/Col 7)
15–19	494 (16.9)	1120	26 (6.4)	16 (7.8)	0.107	120	
20–24	666 (22.8)	1586	40 (9.8)	30 (14.7)	0.206	327	
25–29	711 (24.4)	1962	89 (21.8)	47 (23.1)	0.343	673	
30–34	412 (14.1)	1214	63 (15.4)	29 (14.2)	0.503	611	
35–39	314 (10.8)	1125	65 (15.8)	32 (15.7)	0.664	747	
40–44	202 (6.9)	743	74 (18.1)	31 (15.2)	0.802	596	
45–49	118 (4.1)	483	52 (12.7)	19 (9.3)	0.900	435	
Total	2917 (100.0)	8233	409 (100.0)	204 (100.0)		3508	0.06

MMR, 890/100,000 live births; CI, 504–1281

Estimating MMR for more recent periods, we replicated the analysis for women under age 30. A total of 1871 women aged below 30 years reported 4668 maternal sisters. Of the 4668 sisters reported, 155 are dead and 93 (60%) died of pregnancy and childbearing related deaths. The MMR computed for this age group was 1286 deaths/ 100,000 live births (95% CI, 1026–1545) referring to a period of 7 years before the survey.

## Discussion

The estimated MMR of 890 deaths/100,000 live births (and the estimate for respondents below 30 years of 1286 deaths/100,000 live births) in Kebbi State are higher than the national average of 576 deaths/ 100,000 live births and is equally one of the highest in the world [1]. The estimates again underscore the assertion that highest burden of maternal mortality in Nigeria are from rural northern Nigeria communities [14]. This remains consistently high due to poor and weak health system in the region, in terms of facilities and human resource among other factors. The situation in Kebbi State is more worrisome where utilisation of maternal health services remains very low. Indicators of maternal health services in Kebbi State shows that 71 % of pregnant women did not attend antenatal care; 1135 of the 1247 births were delivered at home and 261 were assisted by traditional birth attendants (TBA) during delivery while another 318 delivered with “no one present” (NOP) [1].

This study provides the first evidence-based baseline data on MMR in Kebbi State as there has never been such study in the State to the best of our knowledge. Our finding is also consistent with similar studies in neighbouring states in northwest Nigeria estimating MMR for the region to be higher than the national average- usually above 1000 deaths/ 100,000 live births [4, 6, 14]. This finding pointed to the fact that if much is to be achieved in the quest for reduction of MMR in Kebbi State, stakeholders need to urgently review the strategies with a renewed tenacity to institute evidence-based interventions to achieve the goal of reduction of MMR in Kebbi State, and Nigeria in general.

Evidence-based interventions geared towards addressing the 3-delays in accessing obstetrics care services such as improving health infrastructures, provision of skilled manpower at the Primary Heath Care (PHC) facilities, community engagement activities. Community engagement activities should include training of TBA on identification of pregnancy and delivery danger signs, sensitization of community and religious leader e,t,c. Further, Emergency Transport Scheme (ETS) should be piloted within communities with highest maternal mortality burden and later scaled-up to cover the entire State.

Furthermore, the MMR of 890/100,000 live births in the study area as computed from the formula was mainly due to high TFR in the study area. Studies have documented several socio-cultural practices sustaining high fertility levels in northern Nigeria [15, 16]. Practices such as early girl marriage (less than 18 years) should be addressed with the right policies and interventions in the State, if the State is to reduce the scourge of high TFR and maternal mortality. Kebbi State is one of the states with the lowest age at first marriage among women (as low as 12 years) [1]. Thus, community engagement activities to address the age at first marriage among women should be prioritised by the government. Interventions that encourages girl child education beyond secondary school like the Bill and Melinda Gates-funded Girls-for-Health (G4H) project in the State should be adequately monitored as it has potentials of further helping to reduce maternal deaths by raising the age at marriage and thus reduce fertility.

Issues around effects of migration and abortion on MMR estimate are very minimal within the study area. Most women migration are intra-state and are usually due to marriages. Pregnancy is also usually within union due to strict adherence to cultural norms and religious tenets in the study area.

However, limitations of this study are those that are generally associated with similar studies using the sisterhood method of estimating MMR. One of such limitation is that the estimates refers to a period of about 11 years from the period of data collection. Secondly, information on place of residence of dead sisters is not available, therefore the location of respondent is used as a proxy for dead sisters’ location. Thirdly, the estimate was from six LGAs (about one-third) out of the twenty-one LGAs in Kebbi State. However, the randomisation in sampling technique addressed the issue of potential sampling bias. Moreover, the health service utilisation level in the sampled communities is similar to that of other areas in the state.

## Conclusion

Our study provided an estimated lifetime risk (LTR) of maternal death which indicates that 1:16 women will have a pregnancy or childbearing related death. Also, the study found MMR of 890 deaths/100,000 live births in selected rural villages in Kebbi State. The findings provide the policy- makers, government and stakeholders with the baseline information on the MMR situation in the State. Our study results underscore the need to urgently address the scourge of high MMR, if Kebbi State and Nigeria in general will achieve the health for all by year 2030 as stated in the Sustainable Development Goals (SDGs).

## Additional file

Additional file 1: Questionnaire. The file is the English version of the questionnaire used for the study. (DOC 34 kb)

## Abbreviations

CI: Confidence Interval
ETS: Emergency Transport Scheme
G4H: Girls for health
LGA: Local Government Area
LTR: Lifetime risk
MMR: Maternal mortality ratio
NOP: No one present
PHC: Primary health care
SDG: Sustainable Development Goal
SMoH: State ministry of health
TBA: Traditional birth attendants
TFR: Total fertility rate
