# Supplementary material for: Maternal mortality ratio in selected rural communities in Kebbi State, Northwest Nigeria
Source: BMC Pregnancy Childbirth. 2018 Dec 21;18:503. doi: 10.1186/s12884-018-2125-2 (PMC6302485; doi:10.1186/s12884-018-2125-2)
Supplement: Supplementary file 1 — Questionnaire. The file is the English version of the questionnaire used for the study. (DOC 34 kb) [file 12884_2018_2125_MOESM1_ESM.doc]

**Questionnaire of sisterhood study in kebbi state**

Select lga:

Enter the community name:

Interviewer consented: Yes/No

Age of respondents:

Education:

Marital status:

| **INFORMATION ON SISTERS AND THEIR MORTALITY** | | | |
| --- | --- | --- | --- |
| 01 | **How many sisters (born to the same mother) have you ever had who were ever-married (including those who are now dead)?** |  |  |
| 02 | **How many of these ever-married sisters are alive now?** |  |  |
| 03 | **How many of these ever-married sisters are dead?** |  |  |
| 04 | **How many of these dead sisters died while they were pregnant, or during childbirth, or during the six weeks after the end of pregnancy?** |  |  |

Q4b. Name of the dead sister

Q4c. Age of ${Q4b} when she died

Q4d. Number of birth ${Q4b} had before she died. 99. Don’t know

Q4e. Number of children ${Q4b}'s had alive before she died. 99. Don’t know

Q4f. Where did ${Q4b} died? 1. At home 2. At the hospital 3. Others (specify)

**Note:**

*Born to the same mother “This is a critical criteria. We want maternal sisters—as is maternal mortality. So if someone has a sister from the same father but different mothers it’s not applicable. These are issues data collectors should understand otherwise they will just collect info on any “sister”. Even if someone has a relation and they just call each other “sister” **but not maternal sisters that is also not correct.**

Ever-married: This means that at the time of death the sister may have been in a marriage or may have been widowed, divorced or separated. But she was married at some point

Died while pregnant, or…” **This is where the definition of maternal mortality comes in**
